# Kaiso differentially regulates components of the Notch signaling pathway in intestinal cells

**DOI:** 10.1186/s12964-017-0178-x

**Published:** 2017-06-21

**Authors:** Shaiya C. Robinson, Kristina Klobucar, Christina C. Pierre, Amna Ansari, Svetlana Zhenilo, Egor Prokhortchouk, Juliet M. Daniel

**Affiliations:** 1 Department of Biology, McMaster University, Hamilton, L8S 4K1 ON Canada; 2 Current address: Department of Biochemistry and Biomedical Sciences, Michael G. DeGroote Institute for Infectious Disease Research, McMaster University, Hamilton, L8N 3Z5 ON Canada; 3 Current address: Department of Life Science, University of the West Indies at St. Augustine, St. Augustine, Trinidad and Tobago; 4 Federal Research Centre of Biotechnology, Russian Academy of Sciences, Moscow, Russian Federation 117312

**Keywords:** Kaiso, Notch, Dll-1, Jagged-1, Intestinal homeostasis, Intestinal cell fates

## Abstract

**Background:**

In mammalian intestines, Notch signaling plays a critical role in mediating cell fate decisions; it promotes the absorptive (or enterocyte) cell fate, while concomitantly inhibiting the secretory cell fate (i.e. goblet, Paneth and enteroendocrine cells). We recently reported that intestinal-specific Kaiso overexpressing mice (*Kaiso* ^*Tg*^) exhibited chronic intestinal inflammation and had increased numbers of all three secretory cell types, hinting that Kaiso might regulate Notch signaling in the gut. However, Kaiso’s precise role in Notch signaling and whether the *Kaiso* ^*Tg*^ secretory cell fate phenotype was linked to Kaiso-induced inflammation had yet to be elucidated.

**Methods:**

Intestines from 3-month old Non-transgenic and *Kaiso* ^*Tg*^ mice were “Swiss” rolled and analysed for the expression of Notch1, Dll-1, Jagged-1, and secretory cell markers by immunohistochemistry and immunofluorescence. To evaluate inflammation, morphological analyses and myeloperoxidase assays were performed on intestines from 3-month old *Kaiso* ^*Tg*^ and control mice. Notch1, Dll-1 and Jagged-1 expression were also assessed in stable Kaiso-depleted colon cancer cells and isolated intestinal epithelial cells using real time PCR and western blotting. To assess Kaiso binding to the *DLL1, JAG1* and *NOTCH1* promoter regions, chromatin immunoprecipitation was performed on three colon cancer cell lines.

**Results:**

Here we demonstrate that Kaiso promotes secretory cell hyperplasia independently of Kaiso-induced inflammation. Moreover, Kaiso regulates several components of the Notch signaling pathway in intestinal cells, namely, Dll-1, Jagged-1 and Notch1. Notably, we found that in *Kaiso* ^*Tg*^ mice intestines, Notch1 and Dll-1 expression are significantly reduced while Jagged-1 expression is increased. Chromatin immunoprecipitation experiments revealed that Kaiso associates with the *DLL1* and *JAG1* promoter regions in a methylation-dependent manner in colon carcinoma cell lines, suggesting that these Notch ligands are putative Kaiso target genes.

**Conclusion:**

Here, we provide evidence that Kaiso’s effects on intestinal secretory cell fates precede the development of intestinal inflammation in *Kaiso* ^*Tg*^ mice. We also demonstrate that Kaiso inhibits the expression of Dll-1, which likely contributes to the secretory cell phenotype observed in our transgenic mice. In contrast, Kaiso promotes Jagged-1 expression, which may have implications in Notch-mediated colon cancer progression.

**Electronic supplementary material:**

The online version of this article (doi:10.1186/s12964-017-0178-x) contains supplementary material, which is available to authorized users.

## Background

The mammalian intestine is a rapidly self-renewing epithelium that undergoes continual turnover every 3–5 days [1, 2]. The mucosa of the small intestine is folded into finger-like projections known as villi [1], which serve to maximize the surface area over which nutrient uptake occurs [3]. Most intestinal epithelial cells (IECs) migrate toward the tip of the villus, where they undergo apoptosis and are shed into the intestinal lumen [4]. The majority of IECs in the intestinal tract consists primarily of absorptive cells known as enterocytes. The remaining cells are categorized as secretory cells and include mucus-secreting goblet cells, hormone-secreting enteroendocrine cells, and anti-microbial Paneth cells [1, 3–5]. Unlike most terminally differentiated IECs, Paneth cells migrate downward toward the base of intervillar crypts, where intestinal stem and progenitor cell populations also reside [3, 6, 7]. The constant turnover of cells within the intestinal epithelium requires strict regulation of the signaling pathways that govern stem and progenitor cell proliferation and differentiation. While several pathways are critical for intestinal homeostasis, the Notch signaling pathway is indispensible for dictating intestinal cell fate decisions [8–13].

Notch receptors are heterodimeric single-pass transmembrane proteins [10, 14–16] with four receptors present in mammals (Notch1–4), each encoded by a separate gene [15]. Active Notch signaling is initiated upon binding of the Notch receptor to the transmembrane Delta/Serrate/Lag2 (DSL) family of Notch ligands, including Delta-like ligand (Dll)-1, Dll-4 and Jagged-1 [15, 16]. Activated Notch then undergoes multiple proteolytic cleavages, which culminates in the cytoplasmic release of the Notch intracellular domain (NICD) fragment [16]. NICD subsequently translocates to the nucleus and interacts with RBPJ (recombination signal binding protein for immunoglobulin kappa J region; also known as CSL – CBF1, suppressor of hairless, Lag2) to transactivate the expression of Notch target genes, most notably, the Hes (Hairy enhancer of split) family of transcriptional repressors [9, 10, 14].

In the intestines, Notch signaling dictates binary cell fate decisions – progenitor cells that lack Notch signaling are fated toward the secretory cell lineage (i.e. goblet, Paneth and enteroendocrine cells), while those with active Notch signaling are fated to become enterocytes [17]. Thus, misregulated Notch signaling in the intestines perturb cell fate decisions. For example, loss of Dll-1 and combined loss of Hes1, Hes3 and Hes5 result in increased goblet, Paneth and enteroendocrine cells [8, 13, 18]. We recently reported that ectopic expression of the poxvirus and zinc finger (POZ)-zinc finger (hereafter, POZ-ZF) transcription factor Kaiso in the intestines of 12-month old mice (*Kaiso* ^*Tg*^) resulted in chronic intestinal inflammation and a significant increase in secretory cell types compared to non-transgenic (NonTg) mice. This finding, coupled with our observation that expression of the Notch target gene *Hes1* was reduced in *Kaiso* ^*Tg*^ compared to NonTg mice, implicated Kaiso as a negative regulator of Notch signaling [19].

Since Kaiso overexpression in 12-month old mice is reminiscent of loss of Notch pathway activity, we sought to further investigate Kaiso’s role in Notch-mediated intestinal homeostasis and cell fate decisions. We found that the Kaiso-induced increase in intestinal secretory cells occurs prior to the onset of chronic intestinal inflammation, suggesting that the secretory cell phenotype does not manifest as a consequence of Kaiso-induced chronic inflammation. Notably, we found that Kaiso inhibits Dll-1 expression in the intestine, and we postulate that this inhibition contributes to the Kaiso-induced increase in secretory cell types. Surprisingly however, we found that Kaiso promotes Jagged-1 expression, which has been previously implicated in colon cancer progression [20–23]. Collectively, these data highlight novel roles for Kaiso in regulating Notch-mediated intestinal homeostasis.

## Methods

### Mouse husbandry of Kaiso^Tg^ tissues

All mice were fed a standard chow diet and maintained in a specific pathogen-free room on a 12-h light/dark cycle. *Kaiso* ^*Tg*^ mice were identified by genotyping using PCR analysis of DNA isolated from ear snips. All PCR primers used are listed in Table 1. All animals were sacrificed by CO_2_ asphyxiation and cervical dislocation.

**Table 1:** List of primer sequences used for genotyping, ChIP-PCR and qRT-PCR

Experiment	Target	Primer sequence 5′-3′
Genotyping	fSV40-Kaiso	ATCATCAAAGCCGGGTGGGCA
	rSV40-Kaiso	TTTTCTACTCTCCATTTCATTCAAGTCCTC
ChIP	DLL1KBS F	AAGCTCTGCAGCTCTCTTGG
	DLL1KBS R	GGCGACTTTCGTTTTCCTC
	JAG1KBS B F	AGCTCTTGTGGCCTCACTTC
	JAG1KBS B R	CCTCAGGCACTACCTCCAGA
	JAG1KBS C F	CCTGAGGGTGTAAGTGATAGGC
	JAG1KBS C R	GAGGAAAGGGAAATGTTGGG
qRT-PCR	mDll-1 F	GCGACTGAGGTGTAAGATGGAA
	mDll-1 R	TCTCAGCAGCATTCATCGGG
	mDll-4 F	GCAAACTGCAGAACCACACA
	mDll-4 R	TGGCTTCTCACTGTGTAACCG
	mNotch1 F	ACAGTGCAACCCCCTGTATG
	mNotch1 R	TCTAGGCCATCCCACTCACA
	mNotch2 F	ACAGTGTTGGCTCCCTGTTC
	mNotch2 R	ATCGTTTACCTTGCCAGCCA
	mHMBS F	GATGGGCAACTGTACCTGACTG
	mHMBS R	CTGGGCTCCTCTTGGAATG
	hDll1 F	AGAAAGTGTGCAACCCTGGC
	hDll-1 R	CACTCTGCACTTGCATTCCCC
	hJag1 F	CGCAAGCGATGTAGATTGAATATT
	hJag1 R	CGCAAGCGATGTAGATTGAATATT
	β-Actin F	CTCTTCCAGCCTTCCTTCCT
	β-Actin R	AGCACTGTGTTGGCGTACAG

### Kaiso null tissues

Kaiso null (*Kaiso* ^*−/y*^) intestinal tissues were kindly provided by Dr. Egor Prokhortchouk. Intestinal tissues were fixed in Carnoy’s fixative (60% EtOH, 30% chloroform, 10% acetic acid) for 16 h at 4 °C, dehydrated and then paraffin embedded for immunohistochemistry [24].

### Cell culture

HCT116, HT29, and SW480 human colon cancer cells were purchased from the American Tissue Culture Collection. All cells were cultured in Dulbecco’s Modified Eagle’s Medium supplemented with 10% fetal bovine serum and 1% antibiotic-antimycotic (Thermo Fisher Scientific). Stable HCT116 and HT29 cell lines containing the retroviral pRS-shKaiso or pRS-shKaiso-scrambled sequence [25], were selected with 2 μg/mL puromycin, while stable SW480 cells were selected with 4 μg/mL puromycin. All cells were grown and maintained at 37 °C, 5% CO_2_.

### Immunohistochemistry

*Kaiso* ^*Tg*^ intestinal tissues were formalin-fixed and paraffin embedded as previously described [19]. Periodic acid-Schiff (PAS) staining was performed by the John Mayberry Histology Facility at McMaster University. Immunohistochemistry (IHC) analysis of all other protein targets was performed as previously described [19], with the following modifications: antigen retrieval for chromogranin A was accomplished by heating tissues in 10 mM sodium citrate, pH 6.0 for 10 min at sub-boiling temperature; retrieval for NICD, Dll-1, Dll-4, Hes1, and Hes5 was accomplished by heating tissues at sub-boiling temperature for 15 min in TE-Tween (Tris EDTA, 0.05% Tween), pH 9.0; and retrieval for lysozyme was performed with 200 μg/mL proteinase K, 50 mM Tris pH 7.4 at RT for 5 min. Tissues were incubated with the following primary antibodies overnight at 4 °C at the indicated dilutions: rabbit anti-lysozyme (Thermo Scientific cat. #PA1–29680; 1:50); rabbit anti-chromogranin A (Abcam cat. #ab15160; 1:500); rabbit anti-Cleaved Notch 1 Val-1744 (Cell Signaling Technology cat. #4147; 1:75); rabbit anti-Hes1 (Cell Signaling Technology cat. #11988S; 1:80); rabbit anti-Hes5 (Abcam cat. #ab65077; 1:125); rabbit anti-Dll-1 (Abcam cat. #ab84620; 1:100); and goat anti-Dll-4 (R&D Systems cat. #AF1389).

### Immunofluorescence

For fluorescent staining of Jagged-1, tissue sections were prepared as described for IHC, however antigen retrieval was accomplished by treatment with 20 μg/mL proteinase K diluted in TE, pH 9.0 for 20 min at 37 °C. Following incubation with primary antibody (rabbit anti-Jagged-1, Santa Cruz cat. #sc-8303; 1:100) and subsequent washes, tissues were incubated with a 1:500 dilution of secondary anti-rabbit Alexa-546 (Life Technologies cat. #A11010) for 2 h at room temperature (RT). Tissues were then washed 3 times for 10 min with 0.05% Tris buffered saline (TBS)-Tween, once for 5 min with TBS, and counterstained with TOTO-3 iodide (Life Technologies cat. #T3604; 1:2000) for 30 min at RT. Excess TOTO-3 was removed and slides were allowed to dry prior to mounting with Prolong® Gold anti-fade reagent (Life Technologies cat. #P10144).

### Protein isolation

#### Intestinal epithelial cell protein isolation

Small intestines were harvested and flushed with ice-cold saline solution (150 mM NaCl, 2 mM imidazole, 0.02% NaN_3_), cut into 1–2 cm pieces and stirred vigorously in ice-cold sucrose solution (12 mM EDTA, 200 mM sucrose, 20 mM KH_2_PO_4_, 78 mM Na_2_HPO_4_, 0.02% NaN_3_) for 2 h at 4 °C. IECs were separated from large tissue segments using a metal strainer, and then pelleted at 200 x g for 10 min at 4 °C. The cell pellet was washed 3 times with sucrose buffer, lysed in Laemmli sample buffer (LSB, 2% SDS, 5% glycerol, 62.5 mM Tris, pH 6.8) containing cOmplete Mini protease inhibitor cocktail (PIC) tablet (Roche cat. #11836153001) on ice for 15 min with periodic vortexing, and denatured by boiling for 5 min.

#### Cultured cell protein isolation

Cells were washed twice with cold 1X phosphate buffered saline (PBS) and pelleted at 1000 RPM at 4 °C for 5 min. Cell pellets were lysed with LSB containing PIC on ice for 15 min with periodic vortexing and denatured by boiling for 5 min. Denatured lysates were centrifuged at 13,200 RPM, 4 °C for 15 min; supernatants were transferred to new pre-chilled tubes and quantified by DC Protein Assay (Bio-Rad cat. #500–0116).

### Myeloperoxidase (MPO) assay

MPO activity of 50 mg of flash frozen ileum was analyzed as previously described [19, 26]. Briefly, tissues were homogenized in 0.5% HTAB (**h**exadecylt**r**imethyl**a**mmonium **b**romide) buffer via sonication at 30 Hz for 4 min, and centrifuged at 12,000 RPM for 15 min at 4 °C. o-dianisidine dihydrochloride solution was added to the homogenates in triplicate in 96-well plates, and absorbance was measured at 450 nm every 30 s for 90 s, in triplicate readings.

### SDS-PAGE and Immunoblotting

Equal amounts of protein of interest were separated by SDS-PAGE and transferred onto a nitrocellulose membrane. Membranes were blocked with 3% milk diluted in 1XTBS for 1 h at RT, with the exception of membranes probed with anti-cleaved Notch1 Val-1744, which were blocked with 5% BSA in 0.1% TBS-Tween20. Membranes were incubated overnight at 4 °C with primary antibodies at the following dilutions: rabbit anti-Kaiso (1:5000); rabbit anti-Cleaved Notch1 Val-1744 (Cell Signaling Technologies; 1:1000); rabbit anti-Dll-1 (Abcam; 1:500); mouse anti-β-actin (Sigma Aldrich cat#. A5441-.2ML; 1:50,000). Membranes were then washed 5 times for 5 min with 1XTBS, incubated with the appropriate HRP-conjugated secondary antibody for 2 h at RT, and then washed 5 times for 5 min with 1XTBS. Proteins were visualized using Clarity Western ECL Substrate (Bio-Rad cat. #170–5060) and images were acquired using the Bio-Rad ChemiDoc.

### Quantitative real-time reverse transcription PCR

Total RNA was isolated using the NucleoSpin RNA kit (Macherey-Nagel cat. #740984.250) according to manufacturer’s protocols. mRNA was converted to cDNA using the SensiFAST cDNA Synthesis Kit, according to the manufacturer’s instructions (Bioline cat. #BIO-65054) and qRT-PCR analysis was performed using the SensiFAST SYBR Hi-ROX Kit, (Bioline cat. #BIO-92020). Gene expression changes were normalized to β-actin (cultured cells) or hydroxymethylbilane synthase (HMBS; intestinal epithelial cells), and quantified using the standard curve method. Statistical calculations were performed using Student’s t-test, and a *p*-value ≤0.05 was considered statistically significant. The primer sequences used are listed in Table 1.

### Chromatin immunoprecipitation (ChIP)

Chromatin was isolated as previously described [27]. For de-methylation studies, cells were treated with 5 μM 5′-aza-cytidine (5′-aza; Sigma Aldrich; cat. # A2385-100MG) every 17 h for 5 consecutive days. Fifteen micrograms of chromatin was precipitated with 8 μg mouse anti-Kaiso 6F monoclonal [28], 4 μg mouse non-specific IgG (Abcam cat. #ab37415) or 4 μg rabbit anti-Histone H3 (Abcam cat. #ab1791) antibodies. Precipitated DNA fragments were resuspended with nuclease-free water and PCR amplified. Primers used for ChIP are listed in Table 1.

### CpG Island prediction

Potential transcription start sites (TSS) were obtained from the Eukaryotic Promoter Database [29]. DNA sequences at the indicated ranges surrounding the TSS were analyzed using the MethPrimer [30] and EMBOSS Cpgplot [31] programs to predict CpG islands.

### Promoter-reporter assays

To create the pGLuc-4XCSL reporter construct, the 4XCSL sites were subcloned from the pGL2-4XCSL-luciferase vector (a gift from Raphael Kopan; Addgene plasmid #41726) [32], into pGLuc-basic (New England Biolabs) using EcoRI and HindIII. The pCAGGS-NICD expression vector was a gift from Nicholas Gaiano (Addgene plasmid #26891) [33]. SW480 and HCT116 cells were seeded in technical triplicate into 6-well dishes at a density of 4 × 10^5^ cells/well. Approximately 12–16 h post-seeding, cells were co-transfected with 1.0 μg pGLuc-4XCSL, 0.5 μg pRS-β-galactosidase, 0.175 μg pCAGGS-NICD, and the indicated amount of pcDNA3-hKaiso using TurboFect™ Transfection Reagent (Thermo Fisher cat. #R0532). Twenty-four hours post-transfection, culture media was assayed for luciferase using the Biolux™ *Gaussia* Luciferase Assay Kit (New England Biolabs; cat. #E3300L), and read on an LB luminometer (Thermo Fisher). Luciferase activity was normalized to β-galactosidase to control for transfection efficiency. The average luciferase values from 3 biological replicates were calculated and plotted as relative fold change against the empty pGLuc-basic vector. Statistical significance was calculated using one-way analysis of variance (ANOVA).

## Results

### Ectopic Kaiso expression decreases Notch signaling and increases secretory cell numbers independent of Kaiso-induced inflammation.

**Fig. 1:**
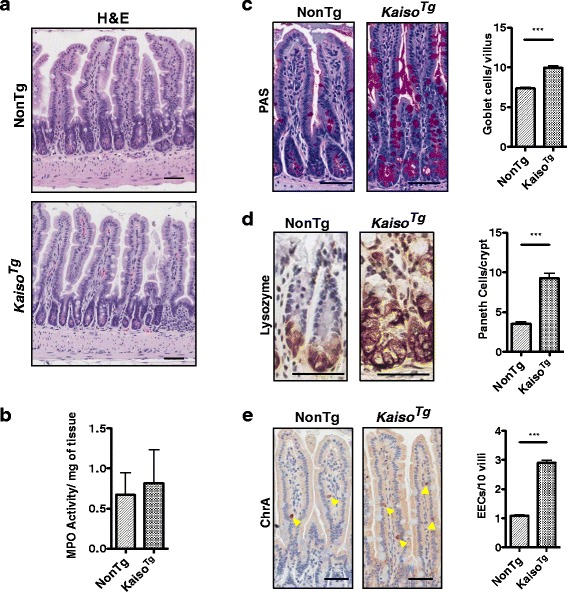
Kaiso phenocopies loss of canonical Notch signaling independently of intestinal inflammation. **a** Hematoxylin & eosin (H&E) staining of 3-month (mo.) old *Kaiso* ^*Tg*^ intestines do not show widespread intestinal tissue damage due to chronic inflammation. **b** Myeloperoxidase (MPO) activity of 3-mo. old *Kaiso* ^*Tg*^ is unchanged relative to age-matched NonTg mice. **c**-**e** 3-mo. old *Kaiso* ^*Tg*^ mice exhibit an increase goblet (PAS - periodic acid Schiff), Paneth (Lysozyme) and enteroendocrine (EEC; ChrA - chromogranin A, yellow arrowheads) cell numbers relative to age-matched NonTg mice. Statistical significance determined using student’s t-test. Error bars are SEM, ***p < 0.0005. Scale bar, 50 μm

Our finding that Kaiso overexpression in the murine gut (*Kaiso* ^*Tg*^) results in chronic intestinal inflammation and an increase in secretory cells in 12-**mo**nth (mo.) old mice compared to age-matched NonTg mice [19], was paradoxical since chronic inflammation is typically associated with a reduction in goblet cells [34, 35]. Thus, we sought to determine whether the *Kaiso* ^*Tg*^ secretory cell phenotype occurred independently, or as a consequence of the Kaiso-induced chronic inflammation by analyzing sub-clinical *Kaiso* ^*Tg*^ mice. Thus, we first analysed the small intestine of 3-mo. old mice for signs of chronic inflammation. The small intestines of 3-mo. old *Kaiso* ^*Tg*^ mice do not exhibit signs of widespread chronic intestinal inflammation visible in 12-mo. old *Kaiso* ^*Tg*^ mice – i.e. extensive villus blunting, thickened submucosa, extensive neutrophil infiltration, etc. [19] (Fig. 1a). Consistent with the lack of inflammation-related tissue damage, there was no significant difference in myeloperoxidase (MPO) activity (a measure of activated neutrophils and a surrogate marker for inflammation) between 3-mo. old NonTg and *Kaiso* ^*Tg*^ mice (*p* = 0.792; Fig. 1b).

Since we did not observe neutrophil-specific intestinal inflammation in 3-mo. old *Kaiso* ^*Tg*^ mice, we next assessed the secretory cell types in these mice. Small intestines were stained with periodic acid-Schiff stain (PAS), or labeled with antibodies against lysozyme and chromogranin A to identify goblet, Paneth and enteroendocrine cells, respectively. We observed a significant increase in all three types of secretory cells (*p* < 0.0001) in 3-mo. old *Kaiso* ^*Tg*^ mice compared to NonTg siblings (Fig. 1c, d and e).

Given that Kaiso overexpression drives an increase in secretory cells, we hypothesized that loss of Kaiso would result in a reduced number of secretory cells. We thus examined age-matched *Kaiso* ^*−/y*^ and NonTg mice for goblet cell numbers using alcian blue. Intriguingly, we did not observe a significant difference in the number of goblet cells (Additional file 1: Fig. S1).

**Fig. 2:**
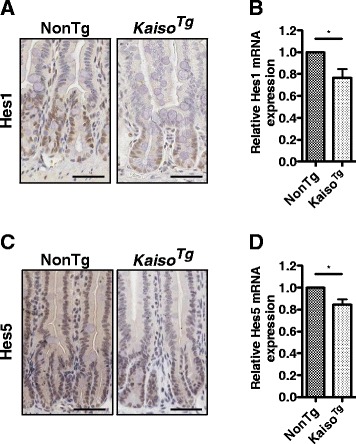
Notch pathway effectors Hes1 and Hes5, are reduced in *Kaiso* ^*Tg*^ mice. Comparison of Hes1 and Hes5 expression in the small intestine of 3-mo. old NonTg and *Kaiso* ^*Tg*^ mice by immunohistochemistry (**a**, **c**) and qRT-PCR (**b**, **d**) demonstrate decreased expression of both Notch effectors in *Kaiso* ^*Tg*^ mice relative to NonTg siblings. Statistical significance determined using student’s t-test. Error bars are SEM, *p < 0.05. Scale bar, 50 μm

Several studies have highlighted the critical role of Notch signaling in mediating intestinal cell fate decisions, by demonstrating that loss of Notch signaling produces an increase in secretory cell types [1, 8–13, 18, 36, 37]. Thus, we examined *Kaiso* ^*Tg*^ intestinal tissues for the expression levels of two downstream Notch pathway effectors, the Hes1 and Hes5 transcription factors, which are known to inhibit secretory cell fates [13]. Indeed, we observed reduced Hes1 and Hes5 expression in *Kaiso* ^*Tg*^ compared to NonTg mice as determined by IHC (Fig. 2a, c). Examination of *Hes1* and *Hes5* transcript levels using mRNA isolated from intestinal epithelial cells (IECs) revealed marginal, but significant, reductions in the relative expression levels of both *Hes1* and *Hes5* in *Kaiso* ^*Tg*^ mice compared to NonTg siblings (Fig. 2b, d). Together, these data suggest that Kaiso overexpression inhibits, but does not completely abolish, Notch pathway activation in intestinal cells. More importantly, our data indicate that Kaiso’s effects on Notch signaling precede the onset of Kaiso-induced intestinal inflammation.

### Kaiso inhibits Notch1 but not Notch2 expression in the small intestine.

**Fig. 3:**
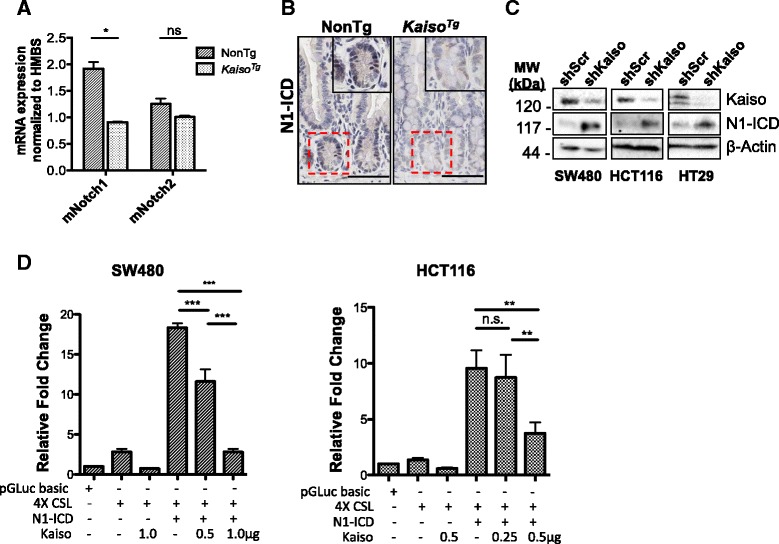
Kaiso inhibits Notch1 expression and N1-ICD levels**. a** qRT-PCR analysis revealed decreased mNotch1 and mNotch2 in isolated IECs of *Kaiso* ^*Tg*^ compared to NonTg mice. **b** IHC revealed reduced N1-ICD protein levels in *Kaiso* ^*Tg*^ mice. Insets are enlarged images of the area boxed with a dotted line. **c** Stable Kaiso-depleted (shKaiso) SW480, HCT116, and HT29 cells exhibit increased levels of N1-ICD as determined by western blot (gamma changes employed). **d** Kaiso overexpression attenuates N1-ICD-mediated transactivation of the 4xCSL artificial promoter in SW480 and HCT116 cells. Cells were co-transfected with 0.5 μg pGLuc-4xCSL, 0.25 μg pCAGGS-N1-ICD, and the indicated amounts of pcDNA3-hKaiso. Statistical significance was determined by student’s t-test or one-way ANOVA with Bonferroni post-test. Error bars are SEM, ns -not significant, *p < 0.05, **p < 0.001, ***p < 0.0001. Scale bar, 50 μm

Since our data suggest that Kaiso overexpression suppresses Notch signaling in the intestine of 3-mo. old mice, we next sought to determine how Kaiso regulates this pathway. Although all four Notch receptors are expressed in the intestinal mucosa, Notch1 and Notch2 are specifically expressed in IECs and are responsible for governing cell fate decisions in the intestines [11, 36, 38, 39]. IECs from 3-mo. old *Kaiso* ^*Tg*^ and NonTg mice were isolated and assayed for *Notch1* and *Notch2* mRNA levels. We observed a significant reduction in *Notch1* mRNA expression in *Kaiso* ^*Tg*^ compared to NonTg mice (*p* = 0.014, Fig. 3a). While *Notch2* mRNA levels were also reduced*,* the change was not statistically significant (*p* = 0.072, Fig. 3a).

Notch pathway activation is characterized by the cytoplasmic release and nuclear translocation of the NICD [40]. Since we observed a reduction in *Notch1* mRNA expression levels, we next assessed Notch1-ICD (N1-ICD) levels in *Kaiso* ^*Tg*^ mice by examining the tissue distribution of N1-ICD in *Kaiso* ^*Tg*^ mice using IHC. As expected, N1-ICD was restricted to the nuclei of crypt epithelial cells in both NonTg and *Kaiso* ^*Tg*^ mice (Fig. 3b). However, *Kaiso* ^*Tg*^ intestinal tissues exhibited an overall reduction in N1-ICD expression compared to their NonTg counterpart (Fig. 3b). To further validate our observations, we examined N1-ICD expression in three stable Kaiso-depleted colon cancer cell lines, as we postulated that loss of Kaiso would result in increased N1-ICD expression. Indeed, N1-ICD expression was increased in stable Kaiso-depleted (shKaiso) SW480, HCT116 and HT29 cells compared to control cell lines that stably express a scrambled shKaiso sequence (shScr) (Fig. 3c).

To examine Kaiso’s effects on N1-ICD function, we performed artificial promoter-reporter luciferase assays using parental HCT116 and SW480 colon cancer cells. HT29 cells were omitted from this analysis since these cells exhibited poorer transfection efficiency compared to HCT116 and SW480 cells. A *Gaussia* luciferase vector containing 4 tandem copies of the *RBP-J/CSL* consensus sequence (pGLuc-4XCSL) was co-transfected with expression vectors encoding N1-ICD (pCAGGS-N1-ICD) and Kaiso (pcDNA3-hKaiso). Co-expression of pCAGGS-N1-ICD and pGLuc-4XCSL resulted in a ~ 15- and ~18-fold increase in pGLuc-4XCSL activity in HCT116 and SW480 cells, respectively (*p* < 0.0001) (Fig. 3d). However, co-expression with pcDNA3-hKaiso attenuated N1-ICD-mediated transactivation of pGLuc-4XCSL in both cell lines, in a dose-dependent manner (*p* < 0.0001) (Fig. 3d). Collectively, these data demonstrate that Kaiso inhibits Notch1 signaling in intestinal cells.

### Dll-1 expression is reduced in Kaiso^Tg^ mice.

**Fig. 4:**
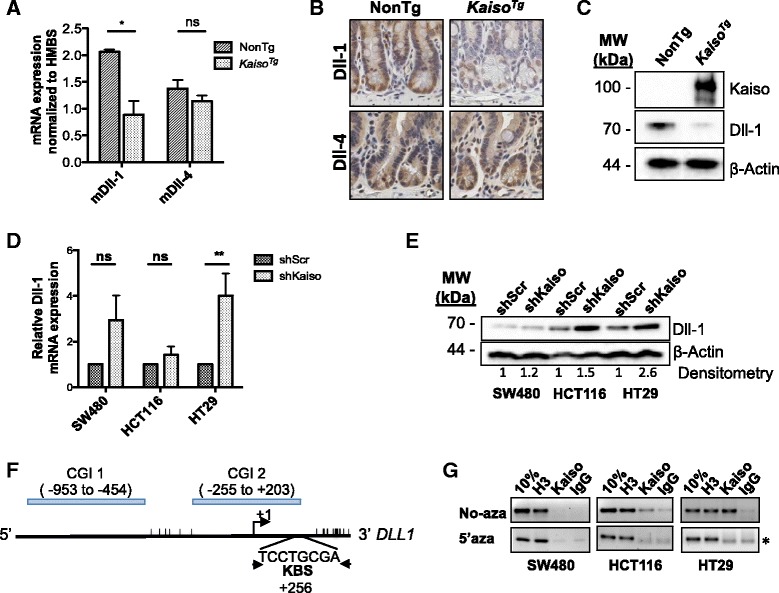
Kaiso inhibits Dll-1 expression in intestinal cells. **a**, **b** Dll-1, but not Dll-4, is reduced in intestinal tissues from *Kaiso* ^*Tg*^ mice compared to NonTg as revealed by qRT-PCR (**a**) and IHC (**b**). **c** Dll-1 protein levels are reduced in intestinal epithelial cells from *Kaiso* ^*Tg*^ relative to NonTg. **d** and **e** Stable Kaiso-depleted (shKaiso) SW480, HCT116 and HT29 colon cells express increased Dll-1 transcripts (**d**) and protein (**e**) compared to controls as determined by qRT-PCR (normalized to β-actin) and western blot analyses, respectively. Western blots were quantified by densitometry (as relative fold change over control). **f** Schematic representation of the human *DLL1* promoter spanning −1000 to +400 bp of the transcription start site (+1). Arrowheads denote regions amplified by PCR; vertical lines – CpG dinucleotides; CGI – CpG island. **g** Kaiso associates with the *DLL1* KBS in HCT116 and HT29 cells, but not SW480 cells. De-methylation with 5’aza-cytidine (5’aza) abolishes Kaiso binding; Histone H3 (H3) and IgG serve as positive and negative controls, respectively. Asterisk (*) denotes primer dimers. Statistical significance was determined by student’s t-test. Error bars are SEM, ns – not significant, **p < 0.005. Scale bar, 50 μm

Previous reports have shown that Notch1 and Notch2 act redundantly in the intestine; thus the single loss of either receptor is insufficient to attenuate Notch signaling [11]. Hence, we surmised that the secretory cell phenotype observed in *Kaiso* ^*Tg*^ mice (Fig. 1) could not be attributed to a reduction in Notch1 expression alone, and that Kaiso may be inducing the secretory cell phenotype via regulation of other components of the Notch pathway. Since Dll-1 and Dll-4 are also implicated in cell fate decisions in the intestine [8], we interrogated the effects of Kaiso overexpression on Dll-1 and Dll-4 expression in our *Kaiso* ^*Tg*^ mice. We first quantified *Dll-1* and *Dll-4* transcript levels by qRT-PCR analysis of harvested IECs from 3-mo. old NonTg and *Kaiso* ^*Tg*^ mice. Interestingly, we observed an ~2-fold reduction in *Dll-1* transcript levels in *Kaiso* ^*Tg*^ mice compared to NonTg siblings (*p* = 0.04), while *Dll-4* transcript levels remained relatively unchanged (*p* = 0.303) (Fig. 4a). We next assessed the in vivo expression levels of Dll-1 and Dll-4 in our *Kaiso* ^*Tg*^ intestines by IHC and found a striking reduction in Dll-1 positive cells in intestines from *Kaiso* ^*Tg*^ mice compared to NonTg siblings (Fig. 4b). Consistent with qRT-PCR analyses, Dll-4 tissue expression was relatively unchanged (Fig. 4b). In support of our findings, western blot analysis also revealed a marked reduction in Dll-1 protein levels in isolated *Kaiso* ^*Tg*^ IECs relative to NonTg (Fig. 4c).

To further validate our in vivo findings, Dll-1 transcript and protein levels were also assessed in stable Kaiso-depleted SW480, HCT116 and HT29 cells. Increased levels of *Dll-1* transcripts were observed in all three Kaiso-depleted cell lines (SW480, HCT116 and HT29) relative to the scrambled controls, but the change in *Dll*-1 mRNA levels was only statistically significant in Kaiso-depleted HT29 cells (*p* = 0.012) (Fig. 4d). Immunoblot analysis of Dll-1 protein expression revealed an ~1.2-, ~1.5- and ~2.6-fold increase in Kaiso-depleted SW480, HCT116 cells, and HT29 cells, respectively (Fig. 4e). Together, these data demonstrate that Kaiso inhibits Dll-1 expression in intestinal cells, and raise the possibility that the observed increase in secretory cell types is due to Kaiso-mediated Dll-1 loss.

### Kaiso associates endogenously with the DLL1 but not the NOTCH1 promoter.

Our group and others have shown that Kaiso possesses dual-specificity DNA-binding abilities, and recognizes and binds both the sequence-specific Kaiso binding site (KBS – TCCTGCNA, core sequence underlined) and methylated CpG dinucleotides [25, 41–44]. Given Kaiso’s effects on the expression of Dll-1 and Notch1 in intestinal cells, we next assessed whether either of these Notch pathway components might be putative Kaiso target genes. Examination of the *DLL1* and *NOTCH1* gene promoters revealed the presence of one or several putative KBS sequences, and numerous CpG dinucleotides. We assayed whether Kaiso associated with these loci in a sequence- or methylation-specific manner. To this end, chromatin was isolated from untreated SW480, HCT116 and HT29 cells, and from cells treated with 5 μM 5′-aza-cytidine for 5 consecutive days. Chromatin immunoprecipitation (ChIP) was performed using the Kaiso-specific 6F monoclonal antibody [28], or with histone H3 and non-specific IgG as positive and negative controls, respectively. The *DLL1* gene promoter spanning −1000 to +400 bp of the transcriptional start site (TSS) contains 2 CpG islands (CGIs; Fig. 4f & Additional file 2: Fig. S2), and has one full KBS located 256 bp downstream of the TSS which is not only surrounded by 6 CpG dinucleotides, but also itself encompasses a CpG dinucleotide, TCCTGCGA (Fig. 4f). Thus, ChIP-PCR was performed on the region encompassing the KBS and surrounding 6 CpG dinucleotides to ascertain whether Kaiso binding occurred in a sequence-specific or methylation-dependent manner. ChIP analysis revealed that Kaiso associated endogenously with the *DLL1* KBS site in untreated HCT116 and HT29 cells, but not in SW480 cells (Fig. 4g). However, Kaiso binding was abolished upon de-methylation with 5′-aza-cytidine (Fig. 4g, 5’aza-treated), suggesting that Kaiso associates with the *DLL1* promoter in a methylation-dependent manner despite the presence of the KBS.

**Fig. 5:**
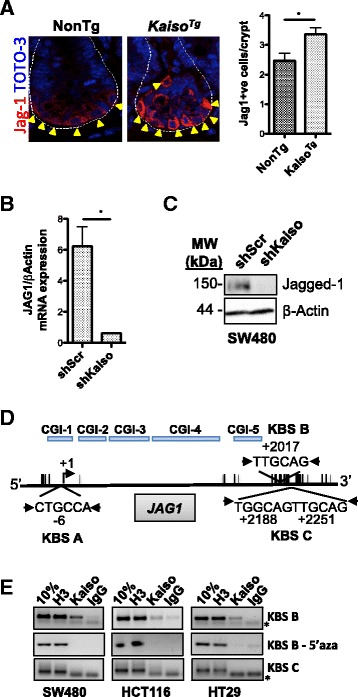
Kaiso promotes the expression of Jagged-1. **a** Immunofluorescence staining revealed enhanced Jagged-1 expression and increased numbers of Jagged-1 positive cells (yellow arrowheads), as quantified in the associated graph, in *Kaiso* ^*Tg*^ crypts. The crypt boundary is indicated with a dotted white line. **b** *Jagged-1* mRNA and (**c**) protein levels are reduced in stable Kaiso-depleted SW480 cells, as determined by qRT-PCR and western blot, respectively. **d** Schematic representation of the human *JAG1* promoter from −200 bp to +2500 bp of the transcription start site (+1). Arrowheads indicate PCR-amplified regions. Vertical lines represent CpG dinucleotides. CpG Islands (CGI) 1–5 are located −112 to +114, +165 to +540, +547 to +824, +853 to +1458, and +1615 to +1886, respectively. **e** Kaiso associates with KBS-B, but not KBS-C of the *JAG1* promoter. 5’aza-treatement led to loss of Kaiso binding to KBS-B. Asterisk (*) denotes primer dimers. Statistical significance determined by student’s t-test. Error bars are SEM, * *p* < 0.05

The *NOTCH1* promoter contains three core KBS sites at positions −1599 bp, −1464 bp and −341 bp of the TSS. However, endogenous Kaiso binding was not observed at any of these loci in the colon cancer cell lines tested (data not shown), despite the presence of multiple CpG dinucleotides in the region analyzed.

### Kaiso promotes expression of the Notch ligand Jagged-1.

Unlike Dll-1 and Dll-4, the Notch ligand Jagged-1 is dispensable for determining secretory cell fate decisions in the intestines [8]. However mounting evidence has highlighted an important role for Jagged-1 in Notch-mediated colon carcinogenesis and progression [20–23, 45–47]. Given that Kaiso promotes polyp formation in the *Apc* ^*Min*/+^ mouse model of colon cancer and reduces overall survival of *Apc* ^*Min*/+^ mice [24, 26], we were prompted to examine Jagged-1 expression and tissue localization in our *Kaiso* ^*Tg*^ mice. Using immunofluorescence staining, we observed Jagged-1 positive cells primarily in the crypts of both NonTg and *Kaiso* ^*Tg*^ mice (Fig. 5a). Notably, Kaiso-overexpression led to enhanced Jagged-1 expression, and an increase in the number of positively stained cells compared to NonTg counterparts (Fig. 5a). Consistent with this finding, loss of Kaiso in stable Kaiso-depleted SW480 cells resulted in reduced Jagged-1 transcript and protein levels compared to control cells (Fig. 5b, c). Notably, HCT116 and HT29 cells do not express significant levels of Jagged-1 mRNA or protein, and thus the effects of Kaiso depletion in these cell lines were negligible (data not shown). Collectively, these data show that, unlike its effects on Notch1 and Dll-1 expression, Kaiso promotes Jagged-1 expression in intestinal cells.

Since we observed a positive correlation between Jagged*-*1 and Kaiso expression in our studies, and Kaiso has also been found to activate expression of some target genes [48–50], we investigated whether *JAG1* might also be a putative Kaiso target. We searched a region spanning −1000 to +2500 bp of the TSS for putative KBS sites and identified at least five core KBS sites. However we focused on the −200 bp to +2500 bp region since this region had a high frequency of putative KBS sites, and also showed binding at 2 loci on the UCSC ENCODE Database [51]. Five putative CGIs and four core KBS sites (i.e. CTGCNA) were identified in the −200 to +2500 region of the JAG1 promoter at positions −6 bp (KBS-A), +2017 bp (KBS-B), +2188 bp and +2251 bp (collectively, KBS-C) relative to the TSS (Additional file 3: Fig. S3, Fig 5d). Primers were designed to PCR amplify each locus, however given the proximity of the +2188 and +2251 KBSs to each other, primers were designed to encompass both KBSs. Kaiso associated weakly with KBS-A in SW480 and HCT116 cells, but not in HT29 cells (data not shown), and no association between Kaiso and KBS-C was detected in any of the cell lines tested (Fig. 5e). However, strong endogenous Kaiso binding was detected at the KBS-B locus in all three cell lines, which was abrogated upon de-methylation with 5′-aza-cytidine (Fig. 5e). Notably, KBS-B and -C both occur outside the CGIs but are both surrounded by several CpG dinucleotides. Thus similar to the *DLL1* KBS, Kaiso associates with the *JAG1* KBS-B locus in a methylation-dependent manner.

In summary, our data demonstrate that Kaiso-mediated regulation of Notch signaling in intestinal tissues occurs via regulation of multiple Notch pathway components. Indeed, our finding that Kaiso associates with the *DLL1* and *JAG1* promoters in a methylation-dependent manner implicates them as putative Kaiso target genes, and suggests that this may be one mechanism by which Kaiso regulates Notch signaling in intestinal cells. Remarkably, Kaiso appears to repress Dll-1 expression, which plays an important role in intestinal cell differentiation, but activates Jagged-1 expression, which is dispensable for cell fate determination.

## Discussion

The highly conserved Notch signaling pathway is fundamental for several biological processes, including binary cell fate decisions in the intestine where it functions to inhibit secretory cell differentiation, while promoting the absorptive cell fate, reviewed in [1, 2, 5]. Herein we demonstrate that the POZ-ZF transcription factor Kaiso, whose overexpression enhances intestinal polyp formation in *Apc* ^*Min/+*^ mice [26] and drives spontaneous intestinal inflammation, regulates the Notch pathway and secretory cell fates in intestinal tissues [19].

During our initial characterization of 12-mo. old *Kaiso* ^*Tg*^ mice, we noticed that *Kaiso* ^*Tg*^ mice exhibited an increase in neutrophil-specific intestinal inflammation [19] (and our unpublished data) and a Notch-depletion phenotype (i.e. increased numbers of secretory cell types). By examining sub-clinical 3-mo. old mice in this study, we found that younger *Kaiso* ^*Tg*^ mice do not display widespread signs of chronic inflammation, but still phenocopy loss of Notch signaling. This finding suggests that the Notch-depletion phenotype in *Kaiso* ^*Tg*^ mice precedes intestinal inflammation (Fig. 1) and may in fact play a role in the pathology of Kaiso-induced chronic inflammation at later ages. Similar findings were observed by Obata et al.*,* who demonstrated that Notch-signaling inhibition via IEC-specific deletion of *Rbpj* (RBP-J^*IEC/Δ*^) resulted in spontaneous inflammation in the colons of these mice [52]. Similar to *Kaiso* ^*Tg*^, RBP-J^*IEC/Δ*^ mice exhibited increased numbers of secretory cells throughout the intestine, as well as augmented neutrophil infiltration in inflamed regions [52].

Notch pathway activation culminates in the expression of the Hes transcription factor family, which function to suppress the secretory cell lineage in the intestinal epithelium [13, 38]. While Hes1-deficiency is sufficient to drive increased secretory cell numbers in immature mice, this effect is counteracted by Hes3 and Hes5 expression after 2 months of age [13]. Indeed, combined loss of Hes1/3/5 resulted in augmented secretory cell numbers compared to loss of Hes1 alone in 2-mo. old mice [13]. Thus, our hypothesis that Kaiso promotes secretory cell differentiation via Notch pathway inhibition is further strengthened by the observation that 3-mo. old *Kaiso* ^*Tg*^ mice exhibit reduced expression of both Hes1 and Hes5 compared to age-matched NonTg siblings (Fig 2). However, qRT-PCR analyses revealed only a modest reduction in mRNA levels of both transcription factors, suggesting that Notch signaling is not completely abolished in *Kaiso* ^*Tg*^ epithelium. This possibility is consistent with our observation that only Dll-1 but not Dll-4, is reduced in *Kaiso* ^*Tg*^ mice.

Using tamoxifen-inducible mouse models, Pellegrinet et al. demonstrated that *Dll-1* ablation was sufficient to cause a mild increase in goblet cells, while loss of *Dll-4* alone did not affect goblet cell numbers [8]. This study was subsequently substantiated by Stamataki and colleagues, who showed that inducible *Dll-1* knock-out led to an increase in enteroendocrine and Paneth cell numbers [18]. Notably however, genetic loss of both *Dll-1* and *Dll-4* produces a more striking phenotype, where the intestinal epithelium undergoes post-mitotic goblet cell conversion [8]. Moreover, while Dll-1 is able to fully rescue loss of *Dll-4,* Dll-4 is only partially able to rescue loss of Dll-1. Thus, the finding that Dll-1, and not Dll-4, is reduced in *Kaiso* ^*Tg*^ mice compared to their NonTg siblings (Fig. 4), is consistent with these previous reports [8, 18], and may explain why 3-mo. old mice exhibit a relatively modest secretory cell phenotype compared to other mouse models of Notch inhibition [8, 11, 53, 54]. Moreover, although we also observed a reduction in Notch1 expression (Fig. 3), the functional redundancy of Notch1 and Notch2 in the intestine [11] suggests that the Notch-depletion phenotype caused by constitutive Kaiso overexpression is due to either a reduction in Dll-1 alone [8], or a combination of both Notch-1 and Dll-1 inhibition. Nevertheless, our findings support a role for Kaiso in secretory cell fate decisions through suppression of Notch signaling

Although Jagged-1 is dispensable for cell differentiation in the intestines [8], increasing evidence supports a role for Jagged-1 in colon cancer [20, 22, 23, 45, 46]. While the enhanced Jagged-1 positivity in *Kaiso* ^*Tg*^ mice was unexpected (Fig. 5), the data is consistent with our previous findings that Kaiso potentiates *Apc* ^*Min/+*^-mediated tumorigenesis [24, 26], and raises the possibility that one mechanism by which Kaiso promotes intestinal tumourigenesis is via Jagged-1 activation.

Several previous studies have demonstrated that Kaiso is capable of binding to DNA using various mechanisms: either in a sequence-specific manner (via the KBS), via methyl-CpG dinucleotides, or a combination of both [25, 27, 41, 42, 49, 55, 56]. The observation that Kaiso’s association with the *DLL1* and *JAG1* promoters is abolished upon demethylation with 5′-aza-cytidine suggests that Kaiso primarily binds to these regions via methyl-CpG, and not KBS-specific, binding mechanisms. While extensive characterization of Kaiso’s binding mechanisms to the *DLL1* and *JAG1* promoters is beyond the scope of this study, it is possible that the presence of a KBS may act to increase the specificity of Kaiso’s association with methyl-CpGs. Indeed, we reported such a phenomenon with the *CCND1* promoter, where Kaiso was found to associate with the +69 KBS in a methylation-dependent manner. Notably however, mutation of the core KBS nucleotides surrounding the methyl-CpG weakened Kaiso’s association with the *CCND1* + 69 KBS site, suggesting that the presence of the KBS acts to strengthen and/or increase the specificity of binding to methyl-CpG sites [25]. Intriguingly, Kaiso did not associate with *JAG1* KBS-C or the *NOTCH1* KBS loci despite the presence of surrounding CpG dinucleotides, a phenomenon that underscores the complexity of Kaiso-mediated transcriptional regulation of its target genes.

While Kaiso overexpression was found to promote the formation of secretory cell types, contrary to our hypothesis, we did not observe a significant change in the number of goblet cells in *Kaiso* ^*−/y*^ mice (Additional file 1: Fig. S1). During their characterization of *Kaiso* ^*−/y*^ mice, Prokhortchouk and colleagues also reported that loss of Kaiso did not produce gross morphological defects, nor did it cause significant alterations to Kaiso-target gene expression [24]. Given that the Kaiso-like proteins, ZBTB4 and ZBTB38 also bind DNA sequences similar to the KBS [43, 57], it is possible that the lack of a secretory cell phenotype in the *Kaiso* ^*−/y*^ mice is due to functional redundancy conferred by these proteins, though this remains to be determined empirically. Nevertheless, the finding that Kaiso overexpression exerts distinct effects on Notch1, Dll-1 and Jagged-1 may still be physiologically relevant, since Kaiso expression is elevated in several diseases, including breast [49, 58], prostate [59], and colon cancer [24, 26], and in some cases of Crohn’s disease (our unpublished data).

## Conclusion

In conclusion, this study describes at least one mechanism by which Kaiso regulates Notch-mediated intestinal homeostasis. Specifically, we demonstrate that Kaiso inhibits the expression of Notch1 and the Notch ligand Dll-1, but enhances the expression of Jagged-1. We postulate that Kaiso-mediated repression of Dll-1 is sufficient to promote the increase in secretory cells observed in our mice. While Kaiso-mediated activation of Jagged-1 in the intestinal epithelium likely does not contribute to the secretory cell phenotype, it is possible that the Kaiso/Jagged-1 interaction contributes to colon cancer progression.

## Additional files

Additional file 1: Figure S1. *Kaiso* ^*−/y*^ mice do not exhibit a goblet cell defect**.** (A) Immunofluorescence staining of *Kaiso* ^*−/y*^ mice confirm Kaiso-depletion in the intestinal epithelium. Intestines were counterstained with DAPI (4, 6-diamidino-2-phenylindole) to label the nuclei. (B) Goblet cells from three 4-week old NonTg and *Kaiso* ^*−/y*^ mice were labeled with alcian blue and quantified. *Kaiso* ^*−/y*^ mice do not exhibit a significant change in goblet cells, as determined by student’s t-test. (PDF 770 kb)

Additional file 2: Figure S2. CpG island prediction of the *DLL1* promoter. The *DLL1* promoter spanning −1000 to +400 bp of the TSS was analyzed for putative CpG islands. This region is GC-rich and contains two potential CpG islands at −953 to −454 bp and −255 to +203 bp of the TSS (+1), respectively. (PDF 73 kb)

Additional file 3: Figure S3. CpG island prediction of the minimal *JAG1* promoter. The *JAG1* gene spanning −200 to +2500 bp of the TSS was analyzed for putative CpG islands. This GC-rich region harbors 5 potential CpG islands. (PDF 51 kb)

## Abbreviations

5′-aza: 5′ aza-cytidine
ANOVA: Analysis of variance
CGI: CpG Island
ChIP: Chromatin immunoprecipitation
CSL: CBF1, suppressor of hairless, Lag2
DAPI: 4, 6- Diamidino-2-phenylindole
Dll: Delta like ligand
DSL: Delta/Serrate/Lag2
Hes: Hairy enhancer of split
H & E: Hematoxylin and eosin
HMBS: Hydroxymethylbilane synthase
HTAB: hexadecyltrimethylammonium bromide
IEC: Intestinal epithelial cells
IHC: Immunohistochemistry
*Kaiso*^*Tg*^: Kaiso transgenic
KBS: Kaiso binding site
LSB: Laemmli sample buffer
Mo.: Month
MPO: Myeloperoxidase
N1-ICD: Notch1 intracellular domain
NICD: Notch intracellular domain
NonTg: Non-transgenic
PAS: Periodic acid Schiff
PBS: Phosphate buffered saline
POZ-ZF: Pox virus and zinc finger – zinc finger
qRT-PCR: Quantitative real time polymerase chain reaction
RBP-J: Recombination signal binding protein for immunoglobulin kappa J region
SEM: Standard error of the mean
TBS: Tris buffered saline
TSS: Transcription start site
