# Supplementary figures and images for: Kaiso differentially regulates components of the Notch signaling pathway in intestinal cells
Source: Cell Commun Signal. 2017 Jun 21;15:24. doi: 10.1186/s12964-017-0178-x (PMC5480165; doi:10.1186/s12964-017-0178-x)

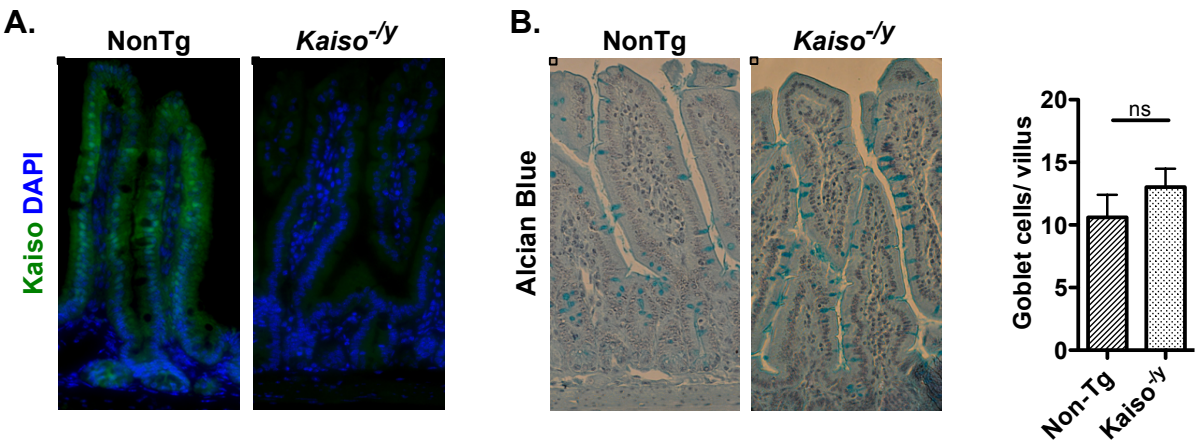

Supplement: Supplementary file 1 — Kaiso −/y mice do not exhibit a goblet cell defect. (A) Immunofluorescence staining of Kaiso −/y mice confirm Kaiso-depletion in the intestinal epithelium. Intestines were counterstained with DAPI (4, 6-diamidino-2-phenylindole) to label the nuclei. (B) Goblet cells from three 4-week old NonTg and Kaiso −/y mice were labeled with alcian blue and quantified. Kaiso −/y mice do not exhibit a significant change in goblet cells, as determined by student’s t-test. (PDF 770 kb) [file 12964_2017_178_MOESM1_ESM.pdf]

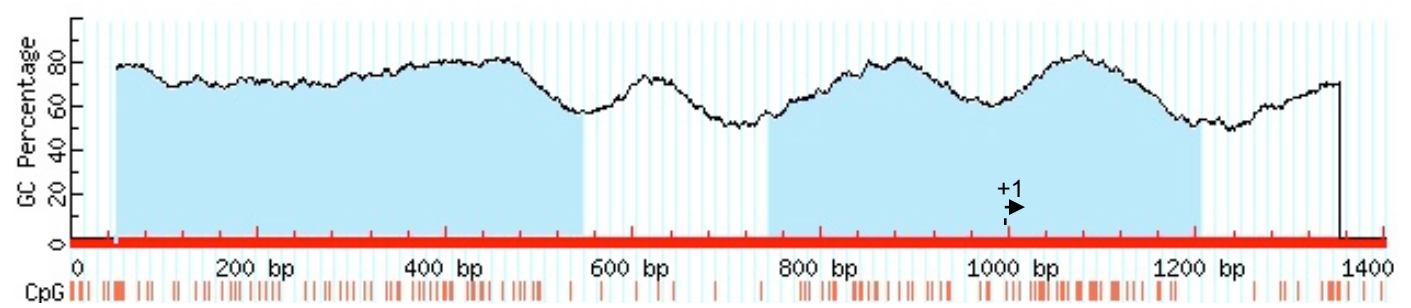

Supplement: Supplementary file 2 — CpG island prediction of the DLL1 promoter. The DLL1 promoter spanning −1000 to +400 bp of the TSS was analyzed for putative CpG islands. This region is GC-rich and contains two potential CpG islands at −953 to −454 bp and −255 to +203 bp of the TSS (+1), respectively. (PDF 73 kb) [file 12964_2017_178_MOESM2_ESM.pdf]

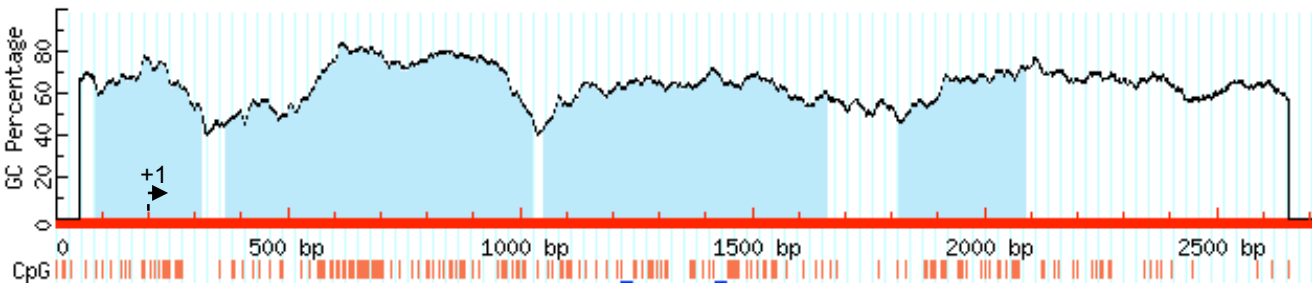

Supplement: Supplementary file 3 — CpG island prediction of the minimal JAG1 promoter. The JAG1 gene spanning −200 to +2500 bp of the TSS was analyzed for putative CpG islands. This GC-rich region harbors 5 potential CpG islands. (PDF 51 kb) [file 12964_2017_178_MOESM3_ESM.pdf]
